# Two sympatric lineages of Australian *Cnestus solidus* share *Ambrosiella* symbionts but not *Wolbachia*

**DOI:** 10.1038/s41437-023-00659-w

**Published:** 2023-11-10

**Authors:** James R. M. Bickerstaff, Bjarte H. Jordal, Markus Riegler

**Affiliations:** 1 Hawkesbury Institute for the Environment, Western Sydney University, Locked Bag 1797, Penrith, NSW 2751 Australia; 2 Australian National Insect Collection, CSIRO, GPO Box 1700, Canberra, ACT 2601 Australia; 3 Museum of Natural History, University Museum of Bergen, University of Bergen, NO-5020 Bergen, Norway

**Keywords:** Phylogenetics, Ecology

## Abstract

Sympatric lineages of inbreeding species provide an excellent opportunity to investigate species divergence patterns and processes. Many ambrosia beetle lineages (Curculionidae: Scolytinae) reproduce by predominant inbreeding through sib mating in nests excavated in woody plant parts wherein they cultivate symbiotic ambrosia fungi as their sole source of nutrition. The Xyleborini ambrosia beetle species *Cnestus solidus* and *Cnestus pseudosolidus* are sympatrically distributed across eastern Australia and have overlapping morphological variation. Using multilocus sequencing analysis of individuals collected from 19 sites spanning their sympatric distribution, we assessed their phylogenetic relationships, taxonomic status and microbial symbionts. We found no genetic differentiation between individuals morphologically identified as *C. solidus* and *C. pseudosolidus* confirming previous suggestions that *C. pseudosolidus* is synonymous to *C. solidus*. However, within *C. solidus* we unexpectedly discovered the sympatric coexistence of two morphologically indistinguishable but genetically distinct lineages with small nuclear yet large mitochondrial divergence. At all sites except one, individuals of both lineages carried the same primary fungal symbiont, a new *Ambrosiella* species, indicating that fungal symbiont differentiation may not be involved in lineage divergence. One strain of the maternally inherited bacterial endosymbiont *Wolbachia* was found at high prevalence in individuals of the more common lineage but not in the other, suggesting that it may influence host fitness. Our data suggest that the two Australian *Cnestus* lineages diverged allopatrically, and one lineage then acquired *Wolbachia*. Predominant inbreeding and *Wolbachia* infection may have reinforced reproductive barriers between these two lineages after their secondary contact contributing to their current sympatric distribution.

## Introduction

Inbreeding through mating with siblings (sib mating) or close relatives can be a successful evolutionary strategy. By reducing genetic recombination, inbreeding can lead to the fixation of advantageous genotypes in populations (Whitlock 2003). Sib mating also effectively removes the constraints of mate finding (Andersen et al. 2012) allowing for efficient reproduction and establishment of propagules in novel environments (Jordal et al. 2001). In insects with haplodiploid sex determination systems, such as in the Hymenoptera or in some lineages of the weevil subfamily Scolytinae, negative impacts commonly associated with inbreeding, such as the accumulation of deleterious alleles, are purged in haploid males (Smith 2000; Peer and Taborsky 2005). Despite the apparent evolutionary advantage of inbreeding, few studies have explored whether and how inbreeding affects species diversification rates. In haplodiploid Scolytinae, regular or predominant inbreeding favours dispersal (Jordal et al. 2000, 2001; Gohli et al. 2016) and, ultimately, promotes allopatric divergence and reinforces reproductive isolation after secondary contact of diverged lineages (Gohli et al. 2017). Recent phylogenetic modelling has furthermore identified predominant inbreeding in Scolytinae as a primary factor responsible for increased diversification rates when compared to outbred Scolytinae lineages (Gohli et al. 2017).

Interactions with microbial endosymbionts such as the common endosymbiont *Wolbachia* (Alphaproteobacteria), which infects 50 to 60% of arthropods species, can contribute to reproductive isolation between arthropod lineages (Brucker and Bordenstein 2012; Weinert et al. 2015; Detcharoen et al. 2019). These intracellular endosymbionts are maternally inherited and can affect host reproduction by inducing a wide array of reproductive manipulations, such as cytoplasmic incompatibility (CI), thelytokous parthenogenesis, male killing and feminisation (Werren et al. 2008; Shropshire and Bordenstein 2016; Kaur et al. 2021). Many of these manipulations promote the production of infected females thereby increasing *Wolbachia* prevalence, and can also influence host population dynamics, sex ratios, sex determination systems and/or genetic diversity. In particular, the intraspecific genetic diversity of other maternally inherited elements, such as mitochondria, can be skewed when a *Wolbachia* strain invades host populations (Hurst and Jiggins 2005; Avtzis et al. 2008; Arthofer et al. 2010; Cariou et al. 2017; Arif et al. 2021; Morrow and Riegler 2021). The reproductive and behavioural host manipulations of *Wolbachia* can also become coupled with other reproductive barriers, promoting reinforcement of reproductive isolation (Telschow et al. 2005; Shropshire and Bordenstein 2016; Cruz et al. 2021; Kaur et al. 2021; Bruzzese et al. 2021). Furthermore, *Wolbachia* can confer fitness benefits in some host species, including increased fecundity, nutrient provisioning and pathogen defence (Zug and Hammerstein 2015).

Many lineages of Scolytinae ambrosia beetles engage in predominant inbreeding by sib mating (Keller et al. 2011; Storer et al. 2017; Johnson et al. 2018). Recent phylogenetic analyses have identified at least eight independent origins of inbreeding in Scolytinae (Jordal and Cognato 2012; Pistone et al. 2018), illustrating the evolutionary success of predominant inbreeding in these beetles (Kirkendall et al. 2015). This mating system has mostly been explored in Xyleborini species which construct nests in the xylem tissue of woody plants and cultivate symbiotic ambrosia fungi from propagules or spores stored in the beetles’ mycetangia (also commonly referred to as mycangia) (Francke-Grosmann 1956; Hulcr and Stelinski 2017; Mayers et al. 2022). All studied Xyleborini species are haplodiploid, wherein mated diploid foundress females produce diploid daughters and haploid sons. These siblings can mate with one another, and fertilised females then leave the natal nest to locate new host substrates and start new families (Takenouchi and Takagi 1967; Kirkendall 1983; Jordal et al. 2000, 2002). Males of Xyleborini species are typically dwarfed, almost blind and flightless, and commonly mate with their sisters; however, in some species, such as *Xylosandrus germanus* and *Euwallacea fornicatus*, they have been observed to leave their natal nest and wander on the bark of the host plant, presumably to mate with females in other nests in the same host tree (Bright 1968; Peer and Taborsky 2004; Keller et al. 2011; Cooperband et al. 2016). Although additional outbred offspring will add to the fitness of the male, such outbred offspring can suffer outbreeding depression, as experimentally observed in *Xylosandrus germanus*, thereby reducing the fitness of the female (Peer and Taborsky 2005).

The ecology and distribution of Xyleborini species in Australia has been scarcely studied despite their diversity, which include 58 species from 20 genera (Pullen et al. 2014). Of these, two *Cnestus* species are commonly collected and widely distributed throughout eastern Australia, from Far North Queensland to Tasmania, *Cnestus solidus* (Eichhoff 1868) and *Cnestus pseudosolidus* (Schedl 1936). Froggatt (1926) stated that *C. solidus* also occurs in Western Australia, and *C. pseudosolidus* has been intercepted on the Noises Islands, New Zealand, in 1978 (Brockerhoff et al. 2003). Furthermore, there are questions regarding the taxonomy of *C. pseudosolidus*. In both its initial description by Schedl (1936) and subsequent taxonomic revision by Dole and Beaver (2008) it was highlighted that it intergrades morphologically with *C. solidus*, with suggestions that the two species names may be synonymous.

Similar to many other xyleborines (Gohli et al. 2017; Johnson et al. 2018) *Cnestus* species are host generalists: *C. solidus* has been recorded from *Diploglottis australis* (Sapindaceae), *Eucalyptus* spp., in particular smooth-barked eucalypts (Myrtaceae), *Macadamia* sp. (Proteceae), *Malus sylvestris* and *Prunus armeniaca* (Rosaceae), and *C. pseudosolidus* from *Araucaria cunninghamii* (Araucariaceae)*, M. sylvestris* and *Prunus* spp. (Rosaceae), and *Mangifera indica* (Anacardiaceae) (Froggatt 1926; Wood and Bright 1992; Mitchell and Maddox 2010). In general, *Cnestus* species are associated with fungi of the genus *Ambrosiella* (Microascales: Ceratocystidaceae) with which they maintain a coarse co-phylogenetic symbiotic relationship (Skelton et al. 2019; Mayers et al. 2020). However, the fungal symbionts of the two Australian *Cnestus* species and their phylogenetic relationships with *Ambrosiella* are still unknown.

In our study, we explored the genetic diversity of Australian *Cnestus* using mitochondrial and nuclear genome markers and assessed whether they form two distinct sympatric species or their two names should be considered synonymous. Furthermore, we cultured and characterised the diversity of their associated ambrosia fungi. Lastly, because we inadvertently detected *Wolbachia* in some *Cnestus* individuals using universal mitochondrial *cytochrome oxidase I* (COI) barcoding primers, we explored the identity, distribution and prevalence of this endosymbiont in host beetle populations and tested whether this could be indicative of any potential contributions to the divergence of Australian *Cnestus* species.

## Methods

### *Cnestus* collections and *Ambrosiella* culturing

**Fig. 1:**
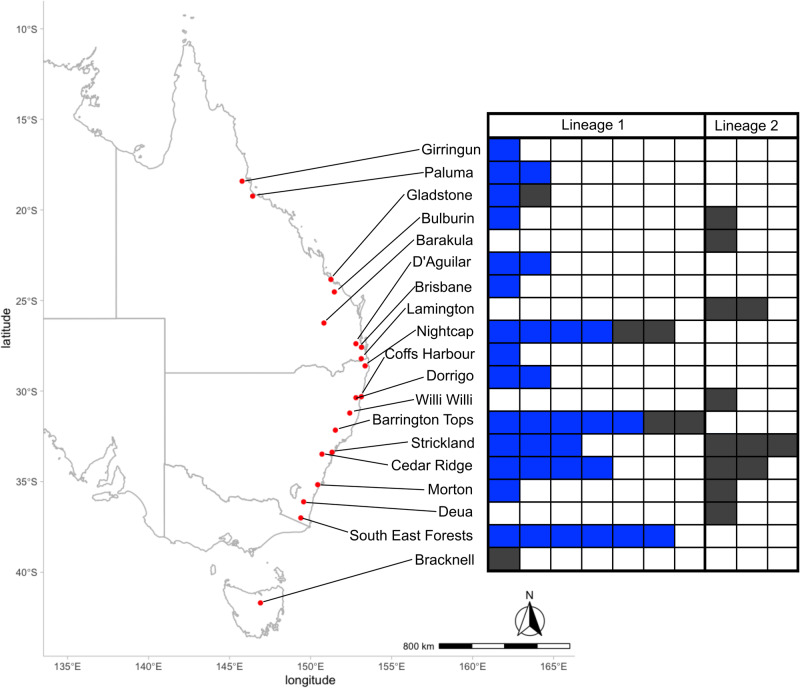
Sampling sites for *Cnestus solidus* in eastern Australia with distribution of individuals belonging to lineage 1 or 2 as shown in filled cells. Lineage assignment occurred based on sequence analysis of mitochondrial and nuclear gene markers. Blue indicates detection of *Wolbachia* and dark grey absence of *Wolbachia* in individuals based on *wsp* PCR assays and sequence analysis.

We analysed a total of 96 individuals of *Cnestus* from 21 localities throughout New South Wales (NSW), Queensland and Tasmania (Fig. 1; Table S1). At 14 sites beetles were collected in flight using cross-vane panel traps (three traps per site, each up to 5 km apart from one another), baited with 70% ethanol as lure, and a 50/50 blend of propylene glycol and pure ethanol in the collection container at the base. In NSW, traps were deployed between September to March throughout 2018–2020, and the propylene glycol/ethanol mix was replaced monthly (weekly at Cedar Ridge Road, Kurrajong, NSW). In Queensland, samples were collected in April 2019 and March 2020. *Cnestus* individuals, once collected, were stored in pure ethanol at −20 °C.

For the remaining seven sites, we used previously collected specimens. Specimens from Lamington National Park were sourced from the Australian National Insect Collection (ANIC); specimens from Brisbane, Barakula State Forest and Gladstone were sourced from the Queensland Department of Agriculture and Fisheries Insect Collection (QDAF), and specimens from Bracknell, Musselroe and Cape Portland were sourced from the Tasmanian Museum and Art Gallery Invertebrate Zoology Collection (TMAG). All these collection specimens had been stored in pure ethanol and kept at −20 °C, except for those from Tasmania, which had been point mounted. As per Schedl (1936) and Dole and Beaver (2008), and by comparisons with the paratype of *C. pseudosolidus*, individuals that were uniformly black and had an abrupt and flattened elytral declivity were identified as *C. pseudosolidus* while those that were red or not uniformly coloured and had a more sloping elytral declivity were identified as *C. solidus*.

*Ambrosiella* was cultured from five live *Cnestus* individuals collected in flight from Cedar Ridge Road (Fig. 1). For this, traps were checked twice daily throughout September and October in 2020 and any newly arrived beetles were removed from the surface of the alcohol mix when still alive. Beetles were split between the pronotum and mesonotum, and the anterior (head and prothorax, approximate location of mycetangia) and posterior (mesothorax, metathorax and abdomen) parts were placed on separate streptomycin malt agar plates (SMA: 1% malt extract, 1.5% agar, 100 ppm streptomycin sulfate added after autoclaving) and incubated at room temperature (Mayers et al. 2018). Fungal colonies were subcultured on SMA every 4-5 days until only one fungal morphotype grew per plate. These morphotypes were then subcultured on plates of malt yeast extract agar (MYEA: 2% malt extract, 0.2% yeast extract, 1.5% agar) to further assess growth and morphology.

### DNA extraction and sequencing

All 96 individuals in this study were used for DNA extraction. Of these, 92 individuals underwent dissections to separate the head, elytra, mycetangia, thorax and abdomen. The head and elytra were stored as tissue vouchers (vouchered at ANIC, with accession numbers listed in Table S1). *Cnestus* beetle DNA was extracted from the thorax and abdomen using the Qiagen PowerSoil kit following a modified protocol; tissues were homogenised in 60 µL of C1 lysis buffer, then 20 µL of proteinase K (20 mg/ml) was added and homogenates were incubated for three hours at 56 °C with gentle shaking. For the mycetangia dissections, the prothorax and the mesothorax were separated with sterilised forceps, and the scutellum was removed from underneath the posterior margin of the pronotal disc. In *Cnestus*, the mesonotal mycetangia are connected to the scutellum (which also harbours mycetangial pits), so both the mycetangia and scutellum were used for symbiont fungal DNA extraction. Mycetangial fungal DNA was extracted using the Gentra Puregene kit following manufacturers protocols optimised for 5–10 mg of tissue. For the four dry specimens (two from Bracknell, one each from Musselroe and Cape Portland), a modified Qiagen DNEasy kit protocol was followed, where the whole specimen was non-destructively lysed in 180 µL of Buffer ATL, then then 20 µL of proteinase K (20 mg/ml) was added and samples were incubated overnight at 56 °C with gentle shaking. Lastly, DNA from the fungal cultures were extracted using the PowerSoil kit protocol described as above.

To assess the genetic diversity of *C. solidus* and *C. pseudosolidus* we amplified fragments of the mitochondrial *cytochrome oxidase I* (*COI*) (*n* = 52) and nuclear arginine kinase (*ArgK*) (*n* = 49) genes from the thorax and abdomen tissue DNA extracts. Initially the LCO1480 and HCO2198 (Folmer et al. 1994) universal *COI* barcoding primers were used; however, in initial tests seven out of 20 individuals preferentially amplified *coxA* gene sequences of *Wolbachia*, as previously observed in other insect *COI* barcoding studies (e.g., Mathenge et al. 2015). Therefore, we used the S1718/A2411 primer pair for amplification and sequencing of *COI* (Simon et al. 1994). Additionally for systematic placement of *C. solidus* and *C. pseudosolidus*, the carbamoyl-phosphate synthetase 2 (*CAD*) (*n* = 5) and elongation factor 1-alpha (*EF1α*) (*n* = 5) genes as well as the 2D-3D segment of the nuclear 28S rRNA (*n* = 7) gene were amplified. To assess the presence of *Ambrosiella* associated with individual specimens, we amplified the internal transcribed spacer *ITS1* and *ITS2* regions of the fungal nuclear *rRNA* gene cassette from all 92 mycetangia DNA extracts using two Ceratocystidaceae-specific primer pairs with overlapping regions for sequence assembly (Harrington et al. 2014). Additionally, the full length *ITS* region (ITS1F/ITS4) was amplified and sequenced from fungal culture morphotypes. For three of the culture morphotypes identified as *Ambrosiella* (through NCBI BLAST), the *28S rRNA* (large subunit; *LSU*), *18S rRNA* (small subunit; *SSU*) and *translation elongation factor 1-alpha* (*tef1-α*) genes were amplified for phylogenetic analyses. Lastly, we screened 92 *Cnestus* individuals (excluding the dried Tasmanian specimens) for *Wolbachia* using primers for the *Wolbachia* surface protein (*wsp*) gene from the thorax and abdomen tissue DNA extracts. For a subset of *wsp*-positive individuals, the multilocus sequence typing (MLST) genes *gatB* (*n* = 7)*, coxA* (*n* = 6)*, hcpA* (*n* = 7)*, ftsZ* (*n* = 3) and *fbpA* (*n* = 8) (Baldo et al. 2006) were sequenced for sequence type identification and phylogenetic analysis of the *Wolbachia* strain. All PCR reactions were performed in 15 µL containing 0.3 µL (20 nM) of each primer (Table S2), 3 µL of 5x MyTaq Red Buffer, 0.3 µL of MyTaq DNA polymerase (Bioline), 2 µL of template DNA (1 µL was used for *COI*) and PCR water (Qiagen). All successfully amplified products were Sanger sequenced in both directions.

Sequence chromatograms were visually assessed for quality. Sequences of protein coding genes were assessed for open reading frames to exclude potential pseudogenes (e.g., nuclear mitochondrial gene copies or NUMTs) in Geneious v10.2.6 (Kearse et al. 2012). For each locus and specimen, forward and reverse complementary sequences were assembled using the de-novo assembly function in Geneious, followed by the removal of primer sequences. Additional sequences were downloaded from GenBank (NCBI), including *LSU, SSU* and *tef1-α* of representative fungal species of the Ceratocystidaceae (Table S3), and *COI, ArgK, CAD* and *EF1α* of *Cnestus* and other scolytine species (Table S4). For comparisons of the fungal *ITS* an aligned reference dataset (Mayers et al. 2020) was downloaded (http://purl.org/phylo/treebase/phylows/study/TB2:S22560). Lastly, *wsp, gatB, coxA, hcpA, ftsZ* and *fbpA* sequences of *Wolbachia* strains characterised in previous scolytine studies (Kawasaki et al. 2010, 2016), and sequences for the *Wolbachia* strains *w*Ha, *w*No and *w*Ri were downloaded from PubMLST (Tables S5, 6).

### Sequence alignment and phylogenetic analysis

All sequences were aligned with MUSCLE using default parameters and then edited manually. To assess the genetic diversity of Australian *Cnestus* species, we analysed *COI* (555 bp) and *ArgK* (750 bp) independently and as a concatenated dataset. For systematic placement of our samples within the *Cnestus* genus, we analysed a combined dataset of the *COI, ArgK, CAD* (462 bp), *EF1α* (813 bp) and *28S rRNA* (741 bp) genes. To assess the phylogenetic placement of *Ambrosiella* sequences obtained from mycetangia DNA extracts and fungal cultures of *Cnestus*, we aligned their *ITS* sequences (555 bp) with the previously aligned *ITS* dataset from Mayers et al. (2020) to preserve diagnostic indel regions. We also used *LSU* (582 bp)*, SSU* (1692 bp) and *tef1-α* (1250 bp) to explore the systematic identity of the *Ambrosiella* cultures obtained from Cedar Ridge Road. All *Wolbachia wsp* and MLST gene sequences from *Cnestus* beetles collected throughout the range were identical, so a single representative sequence for each marker was used for phylogenetic analyses of *Wolbachia*. We then aligned the *wsp* sequence (603 bp) of this strain with those downloaded from PubMLST, and indels were retained. We also aligned the MLST sequences of this strain with those downloaded from PubMLST, generating a final matrix consisting of *gatB* (369 bp)*, coxA* (402 bp)*, hcpA* (444 bp)*, ftsZ* (435 bp) and *fbpA* (429 bp).

Bayesian and maximum likelihood (ML) analyses were performed on all alignments (except for the multilocus *Ambrosiella* dataset for which only Bayesian analyses were conducted), with 1^st^ and 2^nd^ codon positions of protein coding sequences partitioned differently from 3^rd^ codon positions. Bayesian analyses were performed with BEAST2 v2.6.5 (Bouckaert et al. 2014), using an uncorrelated relaxed log-normal clock and a birth-death tree prior, and nucleotide substitution models of partitioned loci were inferred using the bModelTest package (Bouckaert and Drummond 2017) with up to three independent MCMC runs for 100 million generations sampled every 10,000 generations. Independent runs were combined using LogCombiner v2.6.5 (Bouckaert et al. 2014). Convergence to stationarity and effective sample size values of all model parameters were assessed with TRACER v1.7.1 (Rambaut et al. 2018). A maximum clade credibility tree with a 10% burn-in for all analysed datasets was inferred using TreeAnnotator v2.6.0 (Bouckaert et al. 2014). ML analyses were conducted in IQ-Tree 1.6.12 (Minh et al. 2020) with concatenated alignments. Substitution models were estimated with ModelFinder Plus and bootstrap support was calculated with UFBoot (Hoang et al. 2018) with 1000 replicates.

For comparisons with a previous phylogenetic study of *Ambrosiella* (Mayers et al. 2020), maximum parsimony (MP) analysis was used to explore the phylogenetic relationships of *ITS* sequences obtained in our study with those of this previous study using the previously published analytical pipeline (Mayers et al. 2020). Accordingly, analyses were performed in PAUP v4.0a168 (Swofford 2003) as this software allows gaps to be treated as a fifth character, preserving diagnostic indels present in *ITS* (Mayers et al. 2020). A heuristic search was used, starting trees were sampled via stepwise addition and bootstrap support was calculated with 1000 replicates. This alignment contained 555 characters, of which 385 were constant, 34 were variable and parsimony uninformative, and 136 were parsimony-informative. All trees were visualised with FigTree v1.4.4 (Rambaut 2014) and then edited in Inkscape 1.0.

Following phylogenetic analyses, we tested whether *Cnestus* individuals of the two lineages found in our study (lineage 1 *n* = 40, lineage 2 *n* = 12) constituted distinct species with distance-based and tree-based species delimitation methods. We first calculated between group average nucleotide p-distances for *COI* and *ArgK* between *Cnestus* lineages using MEGA 11 (Tamura et al. 2021). For the distance-based analysis, the online implementation of Automatic Barcode Gap Discovery (ABGD) (Puillandre et al. 2012) with the Kimura 2-P model was used to assess barcoding gap distances in both the *COI* and *ArgK* alignments. We then performed a Bayesian implementation of the Poisson tree processes (bPTP) (Zhang et al. 2013) on the ultrametric trees of *COI* and *ArgK* produced with BEAST2 (Figures S1 and S2, respectively). The bPTP analyses run for 100 million generations and sampled every 10,000 generations. The first 10% of trees were discarded as burn-in for both analyses. Following this, intraspecific mitochondrial haplotype diversity of *Cnestus* populations were inferred with a minimum spanning network analysis implemented in PopART (Leigh and Bryant 2015). In order to further analyse patterns of molecular diversity within and between lineages for *COI* and *ArgK* we applied an AMOVA using the pegas package (Paradis 2010) in R. Three non-synonymous nucleotide substitutions were observed between the two lineages in *COI* while all nucleotide polymorphisms in the *ArgK* alignment were synonymous. Therefore, we further explored whether *COI* violated assumptions of neutral evolution with Tajima’s neutrality test in Mega XI (Tamura et al. 2021).

## Results

### Genetic diversity of Australian *Cnestus*

We sequenced a fragment of the mitochondrial *COI* gene from 52, and a fragment of the nuclear *ArgK* gene from 49 Australian *Cnestus* females (Table S4). Alignments of these genes highlight that two main sequence variants are present throughout populations of Australian *Cnestus*. For both genes we did not find any ambiguous sites in sequence chromatograms that could have been indicative of NUMTs or heterozygosity. Furthermore, all *COI* sequences coded for two similar amino acid sequences (however with three non-synonymous substitutions) which were similar to closely related species further indicating that no pseudogenes had been sequenced. Similarly for the *ArgK* nine non-synonymous substitutions were present in the alignment.

**Fig. 2:**
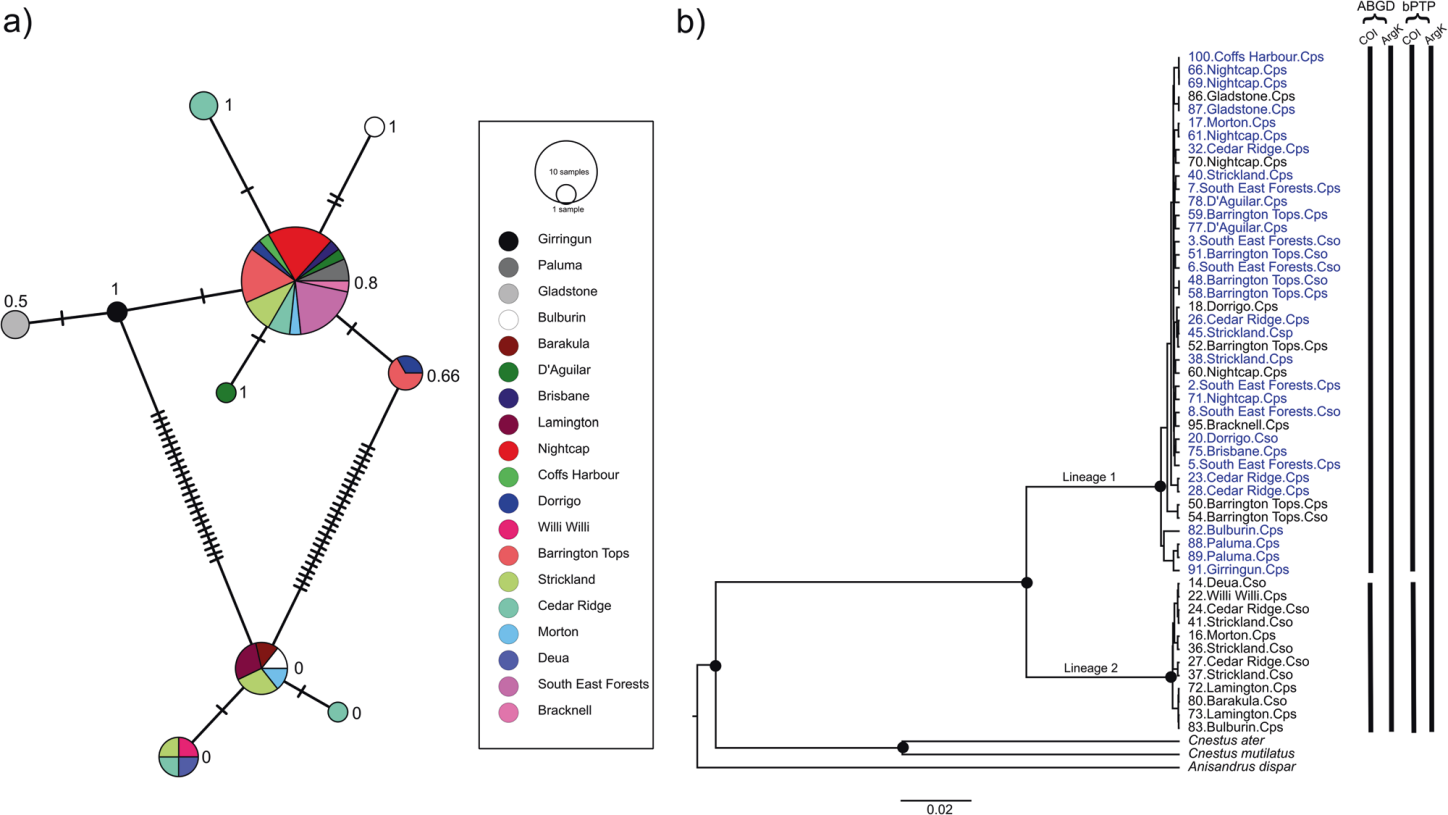
Mitochondrial haplotype and phylogenetic analyses of Australian populations of *Cnestus solidus* spanning their distribution in eastern Australia. **a** Minimum spanning network analysis of the *cytochrome oxidase I* gene fragments of *Cnestus* individuals collected throughout eastern Australia. Dashes on network branches indicate individual nucleotide substitutions between haplotypes and the size of the circles is proportional to the number of samples within each haplotype. Lineage 1 haplotypes are on top and lineage 2 haplotypes on the bottom of the network. Relative proportions of *Wolbachia* infections identified using *wsp* are given next to the circles. **b** Phylogenetic tree of the concatenated *cytochrome oxidase I* (*COI*) and *arginine kinase* (*ArgK*) gene fragments of *Cnestus* individuals collected across eastern Australia. Samples identified as *C. solidus* are labelled Cso, while those identified as *C. pseudosolidus* are labelled Cps. *Wolbachia*-positive individuals are represented in blue. Support is given at the nodes with black dots indicating >0.95 posterior probability and >95% bootstrap. Lineage identity of *Cnestus solidus* is provided on the branches. Scale bar indicates 0.02 substitutions per nucleotide position. To the right of the tips are the results of ABGD and bPTP species delimitation analyses of the *COI* and *ArgK* genes where gaps in the bars represent observed species delimitation splits for *COI* but not *ArgK*.

Bayesian and ML phylogenetic analyses of the concatenated *COI* and *ArgK* gene sequence data sets together with sequences of other xyleborine species (Fig. 2), and of these two genes individually (Figures S1 and S2) produced identical topologies for Australian *Cnestus* indicating no mitochondrial–nuclear discordance. Two distinct sympatric lineages of *Cnestus* were identified and were strongly supported in all analyses (Fig. 2, Figures S1 and S2). We found both lineages distributed from southern NSW to central Queensland (Fig. 1 and Table 1), with a higher abundance of individuals of lineage 1 (*n* = 40) throughout the range compared to lineage 2 (*n* = 12). Furthermore, three specimens of lineage 1 were also found in Far North Queensland, and one in Tasmania. At several sites, individuals of both lineages were collected from the same trap and at the same collection times (Table S1). Between these two lineages we found an average p-distance of 0.00872 for *ArgK* and a substantially higher average p-distance of 0.0912 for *COI*. According to *COI* sequence data, both species delimitation methods identified the two lineages as distinct species; however, based on *ArgK* both lineages were not distinct species.

**Table 1:** Prevalence of the two *Cnestus solidus* lineages, two *Ambrosiella* isolates and one *Wolbachia* strain (as determined by PCR amplification of *wsp*) throughout populations (listed from north to south).

Population	*Cnestus solidus* Lineage 1	*Cnestus solidus* Lineage 2	*Ambrosiella* isolate 1 (Amb1)	*Ambrosiella* isolate 2 (Amb2)	*Wolbachia*
Girringun	1/1	0/1	1/1	0/1	1/1
Paluma	2/2	0/2	2/2	0/2	3/3
Gladstone	2/2	0/2	2/2	0/2	2/3
Bulburin	1/2	1/2	1/1	0/1	1/3
Barakula	0/1	1/1	1/1	0/1	0/2
D'Aguilar	2/2	0/2	2/2	0/2	4/4
Brisbane	1/1	0/1	1/1	0/1	1/1
Lamington	0/2	2/2	1/1	0/1	0/3
Nightcap	6/6	0/6	3/3	0/3	7/12
Coffs Harbour	1/1	0/1	0/0	0/0	1/1
Dorrigo	2/2	0/2	1/1	0/1	3/4
Willi Willi	0/1	1/1	1/1	0/1	0/1
Barrington Tops	7/7	0/7	2/2	0/2	7/12
Strickland	3/6	3/6	2/2	0/2	10/12
Cedar Ridge	4/6	2/6	2/2	0/2	4/13
Morton	1/2	1/2	2/2	0/2	2/3
Deua	0/1	1/1	1/1	0/1	0/2
South East Forests	6/6	0/6	0/5	5/5	12/12
Bracknell	1/1	0/1	1/1	0/1	0/2

Denominator indicates total number of individuals tested per population; different numbers of individuals were tested for *Cnestus*, *Ambrosiella* and *Wolbachia*.

Mitochondrial haplotype network analysis identified two primary haplotypes in each of the two lineages, with a higher haplotype diversity in lineage 1 than lineage 2 (Fig. 2A). Additionally, individuals with unique *COI* haplotypes (singletons) that were one base pair different to more prevalent haplotypes were identified. It is unclear whether these are attributable to small population sample sizes (e.g., Girringun *n* = 1) or whether they represent true mitochondrial diversity within sample sites (e.g., Cedar Ridge *n* = 6 and Bulburin *n* = 2). No significant differences of molecular diversity were identified within lineages for both *COI* (σ = −9.4836e-05, *p* = 0.901) and *ArgK* (σ = −1.7781e-06, *p* = 0.9901). Additionally, *COI* was found to violate assumptions of neutral evolution from Tajima’s Neutrality Test, D = 1.296194 because of the three non-synonymous substitutions. Of these three *COI* non-synonymous substitutions between the two *Cnestus solidus* lineages, only lineage 2 possessed a synapomorphic amino acid with outgroup *Cnestus* species. In addition to the *COI* and *ArgK* analyses of 52 and 49 individuals, respectively, there was an average pairwise similarity between the two lineages for the three nuclear marker genes that were chosen from the overall sample set: 99.6% for *CAD* (*n* = 20), 99.4% for *EF1α* (*n* = 4) and 100% for the *28S rRNA* gene (*n* = 6).

Furthermore, we found no support for Schedl’s (1936) description of *C. pseudosolidus* as a distinct species because morphological species identity did not correlate with genetic lineage identity (Fig. 2B), and, therefore, *C. pseudosolidus* should be considered a synonym of the senior name *C. solidus*. We also inferred the phylogenetic placement of the two *C. solidus* lineages using *COI*, *ArgK*, *CAD*, *EF1a* and *28S rRNA* genes of five individuals relative to five other species within the *Cnestus* genus (Figure S3). All *Cnestus* species formed a strongly supported monophyletic group. Both Bayesian and ML phylogenies identified two distinct and strongly supported lineages of *C. solidus*.

### Isolation of *Ambrosiella* and phylogenetic identification

We cultured three fungal morphospecies from five live *C. solidus* individuals collected at Cedar Ridge. These were identified as an *Ambrosiella* sp., a *Penicillium* sp. and a *Pichia* sp. by *ITS* sequencing and comparison with *ITS* reference sequences using BLAST (NCBI). *Ambrosiella* was cultured from all five plates with the posterior part of beetle specimens (mesothorax, metathorax and abdomen), and from three of the five plates with the anterior part of beetle specimens (head and prothorax), all of which had identical *ITS* sequences. Colonies on MYEA grew to 30 mm in diameter after 14 days at 20 °C and were light green in the centre and darker green toward the edges. Clear hyphae surrounded the fungal mass, and clumped aerial tufts also grew toward the edges of the fungal mass. Cultures smelled sweet and similar to overripe bananas. Amplification and sequence analysis of *ITS* revealed that all cultures had the same *ITS* sequence. Furthermore, we obtained from the cultured *Ambrosiella* isolates the *LSU, SSU* and *tef1-α* sequences which were all identical.

**Fig. 3:**
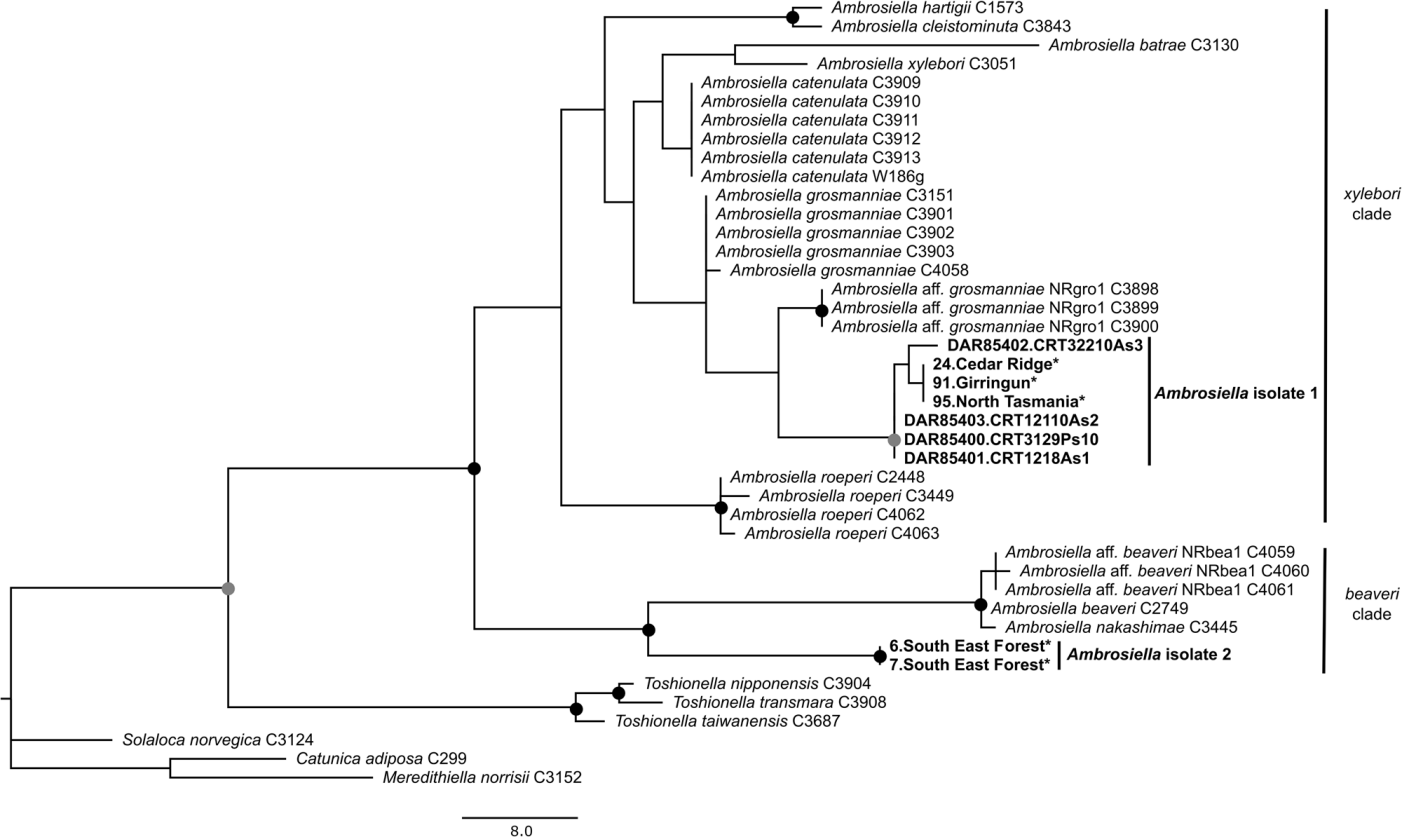
One of 264 most parsimonious trees of ITS sequences (555 bp) of *Ambrosiella* and *Toshonella*, with *Catunica adiposa, Mereditheilla norrisii* and *Solaloca norvegica* as outgroups. Sequences generated from this study are in bold; fungal cultures from *Cnestus solidus* are listed with DAR collection accession codes and original isolation numbers, and sequences directly amplified from beetle mycangia are labelled with an asterisk (*). Bootstrap support is provided at the nodes with black dots indicating >95% support while the grey dot indicates >80% support. Scale bar indicates number of bp changes.

Of the 91 dissected mycetangia DNA extracts, 81 successfully amplified *ITS* using the Ceratocystidaceae primers. A subset of 31 *ITS* PCR amplicons that derived from individuals throughout the range of *C. solidus* were sequenced. All sequences were identified as *Ambrosiella* using BLAST (NCBI), and consisted of two distinct *ITS* sequence variants, representing two different isolates. The most prolific *Ambrosiella* isolate, herein referred to as Amb1, had an *ITS* sequence identical to that of the cultures (Fig. 3), and was found in all populations of both lineages of *C. solidus* except for one population. All individuals collected in South East Forests (SEF), all of lineage 1, were associated with a second *Ambrosiella* isolate with a different *ITS* sequence, herein referred to as Amb2 (Table 1). An attempt was made to collect beetles from South East Forest to culture Amb2, however none were found as most of the habitat was burned during the catastrophic bushfires of 2019–2020.

According to the phylogenetic analysis of *ITS*, Amb1 was placed as a weakly supported sister taxon to *A*. aff. *grossmanniae* in the *A. xylebori* clade (Fig. 3). Amb2 formed a strongly supported group with fungi in the *A. beaveri* clade but was highly diverged from all included species. In the Bayesian multilocus phylogeny, the cultured *Ambrosiella* sp. 1 (Amb1) was placed as a new sister lineage to *A. catenulata*, but with little support (Figure S4). Attempts were made to also obtain *LSU, SSU* and *tef1-α* for Amb2 from mycetangia DNA extracts of South East Forest individuals, however, these PCR reactions failed to amplify.

### *Wolbachia* infection prevalence and strain identification

**Fig. 4:**
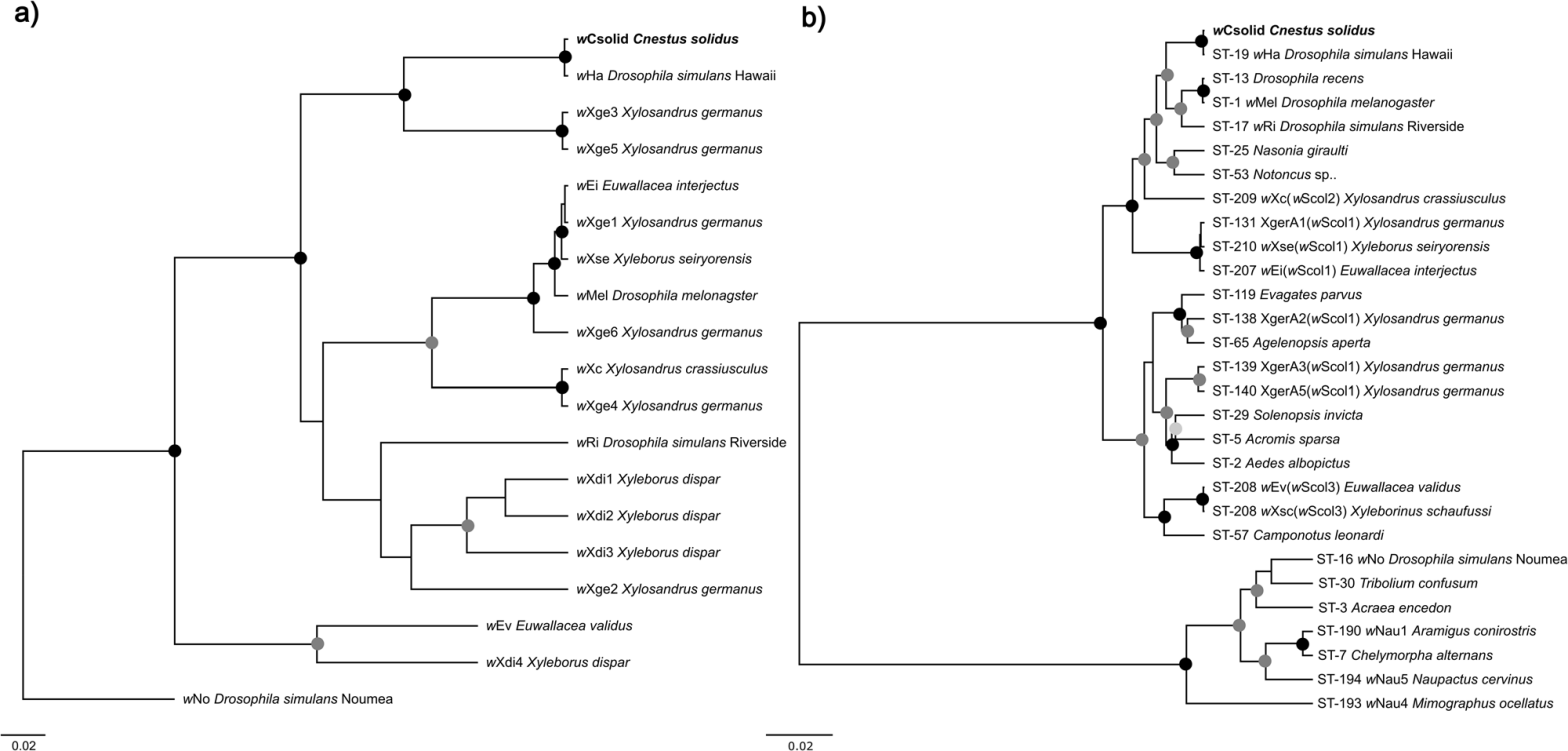
Phylogenetic trees of *Wolbachia* strains found in Scolytinae and other insects. Phylogenies are based on **a** *wsp* gene sequences (603 bp) and **b** *Wolbachia* MLST gene sequences (2079 bp). For all analyses support is provided at the nodes; black dots indicate both >0.95 posterior probability and >95% bootstrap support, dark grey dots indicate only >0.95 posterior probability, and light grey dots indicate only >95% bootstrap support. The *Wolbachia* strain infecting *Cnestus solidus* lineage 1 (*w*Csolid) is in bold. *Wolbachia* supergroup B sequences (*w*No and the strains in the bottom clade in the MLST gene tree) were used as outgroups. Scale bars indicate 0.02 substitutions per nucleotide position.

Of the 93 tested *C. solidus* individuals, 58 were PCR positive for the *Wolbachia* gene *wsp* (Table 1). All *Wolbachia*-positive individuals belonged to lineage 1 (36 *Wolbachia* positive out of 40 individuals), whereas all lineage 2 individuals (12) tested negative. We sequenced *wsp* from 17 individuals and *Wolbachia* MLST genes from five individuals throughout the range of *C. solidus*, and all sequences for each of the genes were identical. Additionally, sequence chromatograms of *wsp* and all MLST genes had no ambiguous sites, suggesting individuals are infected by the same strain belonging to the *Wolbachia* supergroup A, herein referred to as *w*Csolid. Bayesian and ML analyses of all sequence sets identified *w*Ha as the closest relative (Fig. 4a and b), with just one divergent nucleotide between the *wsp* sequences of the two strains, whereas all MLST loci were identical between *w*Csolid and *w*Ha (ST-19).

## Discussion

### Lineage divergence in *Cnestus solidus*

We discovered two genetically distinct yet morphologically indistinguishable lineages of *C. solidus* that occur in sympatry throughout eastern Australia. Both lineages are monophyletic and apparently share the same ecological niche (Froggatt 1926; Wood and Bright 1992; Mitchell and Maddox 2010) which could allow occasional cross-fertilization between family units. Yet, we did not detect any nuclear–mitochondrial discordance between the two lineages. Therefore, these two lineages could perhaps be considered incipient species or even distinct cryptic species which may be unable to hybridize. Furthermore, we detected substantially greater variation in the mitochondrial gene *COI* (9%) than in nuclear markers such as *ArgK* (0.9%), including no substitutions in the 28S rRNA gene. Comprehensive analyses conducted across most of the genera of Xyleborini by Cognato et al. (2020) identified that a 10–12% sequence nucleotide divergence for the mitochondrial *COI* gene and a 2–3% sequence nucleotide divergence for the nuclear *CAD* gene can confidently delimit species boundaries. In contrast to the mitochondrial *COI* nucleotide sequence divergence, the observed nuclear gene sequence divergence between the two *Cnestus* lineages and the results of species delimitation analyses of *ArgK* indicate that they do not constitute distinct species. Furthermore, these patterns are in line with intraspecific divergence within other xyleborine species (Jordal and Kambestad 2014; Gohli et al. 2016; Cognato et al. 2020; Jordal and Tischer 2020; Smith and Cognato 2022). For mitochondrial markers such as *COI*, barcoding sequence divergence of 2–3% (Ashfaq et al. 2016; Hebert et al. 2003) may indicate species boundaries in some insect groups, however, this is not the case for most beetles, and particularly not for inbreeding xyleborine ambrosia beetles (Cognato 2006; Cognato et al. 2020; Smith and Cognato 2022). As discussed by Cognato et al. (2020), haplodiploid female sex ratio biases and inbreeding can greatly amplify the rate at which *COI* nucleotide substitutions occur. While species delimitation analyses of *COI* suggested that the two lineages of *Cnestus* could be separate species, we also found that the *COI* sequence divergence violates assumptions of neutral evolution. This could be due to a selective sweep or population bottleneck in association with *Wolbachia* infection in lineage 1. *Wolbachia* and other maternally inherited factors that are coinherited with mitochondria, influence host fitness and hence prevalence in hosts can cause impact such divergence patterns due to their maternal coinheritance (Cariou et al. 2017; Arif et al. 2021). Therefore, we do not consider these two lineages as distinct species (Templeton 1989; De Queiroz 2007; Freudenstein et al. 2016). Our results further indicate that *C. pseudosolidus* is synonymous with *C. solidus* which is the senior species name. Our finding provides genetic support for a previous study which suggested that the two species appear to intergrade morphologically (Dole and Beaver 2008). We present their synonymy after the concluding remarks.

### Microbial symbionts associated with Australian *Cnestus*

Few studies have detected more than one species, and often just one strain of ambrosia fungi associated with individual ambrosia beetle species. The large, well-developed mycetangia of Xyleborini beetles have been proposed to maintain species-specific symbioses with individual *Ambrosiella* species (Harrington et al. 2014; Mayers et al. 2015). For example, phylogeographic analyses of the *Ambrosiella* associated with *X. germanus* and *Anisandrus dispar* found no genetic diversity in their fungal associates in Europe, supporting the idea of strict clonal transmission in their host beetle species (van de Peppel et al. 2018). Recently, however, multiple closely related *Ambrosiella* species were found in various populations of *X. germanus* throughout its native range in Japan (Ito and Kajimura 2017). In contrast, we detected two phylogenetically distinct *Ambrosiella* isolates that were placed in two separate *Ambrosiella* clades in association with *C. solidus* throughout the range of this beetle species (albeit one of these two *Ambrosiella* isolates was only found in one of the 19 analysed sites). This suggests that symbioses between ambrosia beetle species and fungi may be more flexible than previously anticipated.

The variation that we found in the *Ambrosiella* associated with an ambrosia beetle species is, to our knowledge, the first detection of a clade level symbiont switch in a xyleborine ambrosia beetle species with respect to *Ambrosiella* fungi. This suggests that tight specificity between ambrosia partners may not always be the rule (Harrington et al. 2014; Mayers et al. 2015). Previous co-phylogenetic analyses by Skelton et al. (2019) have identified a rather coarse relationship between *Cnestus* and *Ambrosiella* fungi in the *beaveri* clade. Despite a co-phylogenetic relationship, it was nevertheless demonstrated experimentally that ambrosia beetles can survive on ambrosia fungi of the alternate (*xylebori*) clade and acquire their spores in their mycetangia, even though the probability for spore transfer was low (Skelton et al. 2019). Our data identified that *C. solidus* is predominantly associated with a different (*xylebori*) clade of *Ambrosiella*, suggesting that the symbiosis between ambrosia beetles and fungi can be flexible. It is interesting that we have only identified one population in association with an *Ambrosiella* species of the *beaveri* clade which is usually the main associate with *Cnestus* (Skelton et al. 2019). Future studies should therefore explore in more detail the population genetics and evolutionary history of ambrosia fungi associated with the Australasian species of *Cnestus*. This could yield interesting insights into the observed asymmetric frequency of *Ambrosiella* species associations and reveal any potential selective sweeps that may have generated the current affiliations. For this, however, the culturing and sequencing of other marker genes of Amb2 are needed as currently our results are based on the phylogenetic analysis of *ITS*, a multicopy marker in the fungal genome (Yang et al. 2018). The level of divergence observed in this study could be a result of *ITS* marker divergence and may not reflect the true evolutionary divergence of the isolate. Therefore, further sequence data for the phylogenetic placement of Amb2 are needed.

We also discovered that lineage 1 of *C. solidus* was associated with a new *Wolbachia* strain*, w*Csolid. According to *wsp* gene and MLST analyses, this strain is closely related to *w*Ha, first described from a Hawaiian population of *Drosophila simulans* (Poinsot and Mercot 1997, Ellegaard et al. 2013), and unrelated to *Wolbachia* strains infecting other scolytine beetles (Kawasaki et al. 2016). *w*Csolid was highly prevalent in lineage 1 of *C. solidus*, infecting 34 out of 40 beetles (85%), but entirely absent from lineage 2. *Wolbachia* has been found to infect a wide range of scolytines (Vega et al. 2002; Zchori-Fein et al. 2006; Arthofer et al. 2009; Schebeck et al. 2018; Bykov et al. 2020), and, specifically, haplodiploid ambrosia beetles (Kawasaki et al. 2010, 2016). Kawasaki et al. (2016) suggested that regular inbreeding and haplodiploidy may promote more efficient maternal transmission of this endosymbiont, and this could also contribute to the observed high prevalence of *Wolbachia* in lineage 1 of *C. solidus*.

### Potential involvement of symbionts in *Cnestus* lineage divergence

Allopatric divergence coupled with predominant inbreeding are important factors in scolytine species diversification, while host-tree switching has been found to have little impact on diversification rates (Gohli et al. 2017). According to available information and findings of our study, there appears to be no ecological niche differentiation between the two sympatric lineages of *C. solidus*. First, it has been noted that *C. solidus* is a tree generalist (Froggatt 1926; Wood and Bright 1992; Mitchell and Maddox 2010), however this does not preclude the potential existence of two lineages with some specialisation because we were not able to test host plant associations of our genotyped specimens. Secondly, and more importantly, both lineages were associated with the same *Ambrosiella* fungus. These symbionts may allow an ambrosia beetle to be flexible with respect to host tree selection (Kirkendall et al. 2015; Raffa et al. 2015; Gohli et al. 2017). Additionally, as both lineages were collected in the same traps at the same time at several sites, there appears to be no phenological differentiation in adult emergence and dispersal. Therefore, given that the two *C. solidus* lineages have a likely broad host tree selection, a similar distribution and phenology, as well as the same *Ambrosiella* symbiont, we do not expect niche specialisation or displacement to have generated sympatric divergence between lineages.

Given that *Wolbachia* occurred only in lineage 1, and at high prevalence, this suggests that it may affect the host’s reproduction and/or fitness, and as a consequence possibly also the divergence of the two lineages. Cytoplasmic incompatibility is one of the most frequently reported host reproductive manipulations by *Wolbachia* (Werren et al. 2008; Brucker and Bordenstein 2012; Shropshire and Bordenstein 2016; Kaur et al. 2021). In the case of CI, should an infected male of lineage 1 attempt to outbreed with an uninfected female of lineage 2, this would result in embryonic mortality. However, infected lineage 1 females could potentially still successfully mate with lineage 2 males, leading to a disruption of the current linkage of nuclear genes with mitochondrial genes and *Wolbachia* infections, yet we did not observe this. Therefore, it is unlikely that CI is involved as a mechanism of reproductive isolation between the two lineages unless there is another barrier which prevents successful pairings of lineage 1 females with lineage 2 males. It is also unlikely that *Wolbachia* causes thelytokous parthenogenesis in the infected lineage as we would expect all individuals of the infected lineage to carry *Wolbachia*, which we have not observed. Furthermore, *Wolbachia*-induced thelytoky has so far not yet been demonstrated in any beetle species. We also do not expect that *Wolbachia*-induced male killing or feminisation occurs in *C. solidus*. This is because *Wolbachia*-induced male killing could be disastrous for inbreeding lineages; furthermore, all known and rare examples of *Wolbachia*-induced feminisation are restricted to terrestrial isopods as well as two butterfly and one hemipteran species (Kageyama et al. 2012).

In the absence of any reproductive manipulation, *Wolbachia* could still provide a fitness benefit to lineage 1, and consequently occur at high prevalence in the more commonly found lineage and influence mitochondrial diversity patterns of this species. In some host species *Wolbachia* can provide fitness benefits such as an increase in fecundity, nutrient provisioning and pathogen defence (Zug and Hammerstein 2015). In a haplodiploid scolytine, the date-seed beetle *Coccotrypes dactyliperdia, Wolbachia* was found associated with increased dispersal of infected beetles (Tremmel et al. 2020). Future research should further investigate whether *Wolbachia* manipulates reproduction and/or improves fitness in *C. solidus*.

Furthermore, we have also identified haplotype diversity within the infected lineage of *C. solidus*, suggesting that no recent mtDNA sweeps associated with *Wolbachia* had occurred and *Wolbachia* had been present for long enough for the lineage to acquire new haplotype diversity. Beyond this we found patterns for non-neutral molecular evolution in *COI* that correlates with *Wolbachia* infections in lineage 1. Interestingly, while the infection of *Wolbachia* in lineage 1 did not impact population genetic diversity, we found that the sequence divergence between lineage 2 and outgroup *Cnestus* taxa was smaller than that between lineage 1 individuals and outgroup *Cnestus* taxa. This could indicate independent historical selective sweeps or, alternatively, population bottle necks in both lineages.

In the absence of factors that indicate sympatric speciation processes for the two *C. solidus* lineages, it is likely that they diverged in allopatry followed by secondary contact leading to their contemporary sympatric distribution. It has previously been discussed that sib mating promotes dispersal of propagules into novel environments and subsequent allopatric divergence of lineages (Eliassen and Jordal 2021; Jordal et al. 2000, 2001, 2006). Range expansions and contractions of the Australian wet forests occurred during the Pliocene and this resulted in allopatric separations of many species (Gallagher et al. 2003; Martin 2006; Byrne et al. 2011). It is likely that beetles of lineage 1 became infected with *Wolbachia* when populations were allopatrically separated. Then, after secondary contact, the reproductive isolation of the two lineages has been maintained through sib mating and, possibly, *Wolbachia*-induced host effects.

## Conclusions

Our study represents a comprehensive study of the evolutionary ecology of an Australian scolytine ambrosia beetle species and its associated microbial symbionts. Molecular phylogenetics of the *Cnestus* specimens sampled throughout their range in Australia supported the findings by Dole and Beaver (2008) that *C. solidus* and *C. pseudosolidus* intergrade morphologically and, therefore, constitute only one species. Interestingly, however, we identified two sympatric cryptic lineages with no indication of hybridisation between the two. In the more prevalent beetle lineage 1, we also discovered a new strain of *Wolbachia* that may be associated with the different relative abundance and divergence between the two lineages. Lastly, we also identified two divergent isolates of *Ambrosiella* associated with *C. solidus*, one of which was prevalent throughout their geographical range, and divergent from the *Ambrosiella* symbionts of other *Cnestus* species across the world. These two *Ambrosiella* isolates associated with *C. solidus* highlight a potentially relaxed selectivity of mycetangia towards fungal symbionts. Further investigation of the mitochondrial and nuclear population genetics of *C. solidus* should explore the historical demography of both lineages and look for signatures of effective population size expansion or contraction associated with selective sweeps either caused by, or correlated with, *Wolbachia*.

### Taxonomy

#### *Cnestus solidus* (Eichhoff 1868), comb. nov.

*Xyleborus solidus* Eichhoff 1868: 151

*Xylosandrus solidus* (Eichhoff 1868): combination by Wood and Bright 1992: 800

*Xyleborus pseudosolidus* Schedl 1936: 530, **syn. nov**.

*Xylosandrus pseudosolidus* (Schedl): combination by Wood and Bright, 1992

*Cnestus pseudosolidus* (Schedl): combination by Dole and Beaver 2008

Our phylogenetic analysis of Australian *Cnestus* species did not support Schedl’s (1936) description of *C. pseudosolidus* but provided genetic evidence for the findings by Dole and Beaver (2008) that *C. solidus* and *C. pseudosolidus* intergrade morphologically. Furthermore, we detected both *C. solidus* and *C. pseudosolidus* morphotypes in both genetic lineages. Given the morphological overlap between *C. solidus* and *C. pseudosolidus* (Dole and Beaver 2008; Schedl 1936) and the lack of genetic divergence between these two morphospecies we synonymise the species name *C. pseudosolidus* (Schedl 1936) with the senior *C. solidus* (Eichhoff 1868).

### Supplementary information

Supplementary Figures

Table S1

Table S2

Table S3

Table S4

Tabel S5

Table S6

## Data Availability

Isolates of *Ambrosiella* sp. 1 were deposited with the Plant Pathology Herbarium, Department of Primary Industries NSW (accession codes DAR85400-DAR85403). All *Cnestus* voucher specimens, stored in ethanol and pinned, have been deposited in the ANIC, CSIRO, under accession numbers 25-086124 - 25-085827. All sequence data generated in this study are available from NCBI GenBank with accession codes provided in the supplementary information.
