# Supplementary material for: Two sympatric lineages of Australian Cnestus solidus share Ambrosiella symbionts but not Wolbachia
Source: Heredity (Edinb). 2023 Nov 10;132(1):43–53. doi: 10.1038/s41437-023-00659-w (PMC10798974; doi:10.1038/s41437-023-00659-w)
Supplement: Supplementary file 1 — Supplementary Figures [file 41437_2023_659_MOESM1_ESM.docx]

## Supplementary Information

**
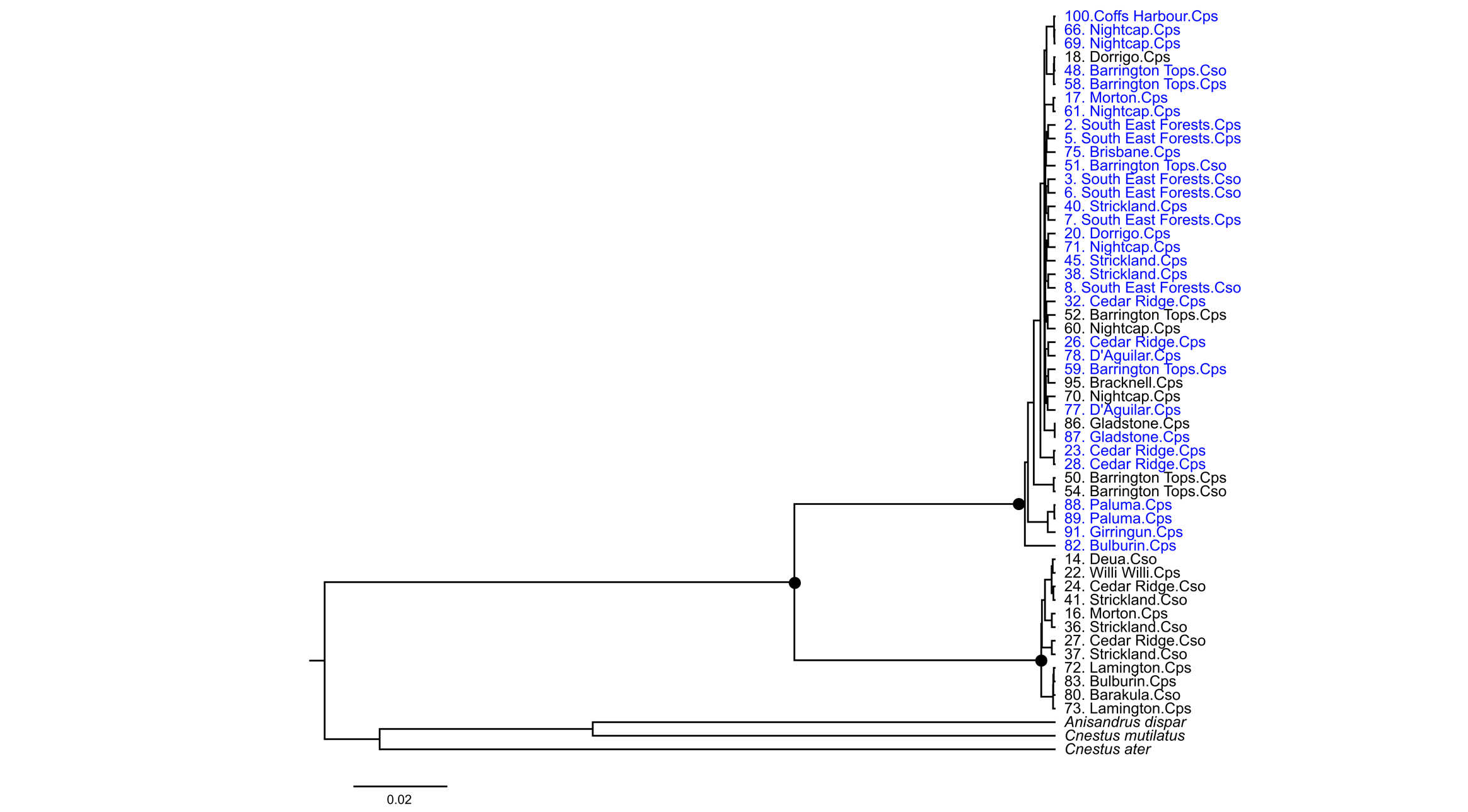
Figure S1.** Phylogenetic tree of the partial *cytochrome oxidase I* gene (555 bp) of *Cnestus solidus* (Cso) and *C. pseudosolidus* (Cps) individuals collected throughout eastern Australia. *Wolbachia*-positive individuals are in blue. Support is provided at the nodes with black dots indicating >0.95 posterior probability and >95% bootstrap support. Scale bar indicates 0.02 substitutions per nucleotide position.


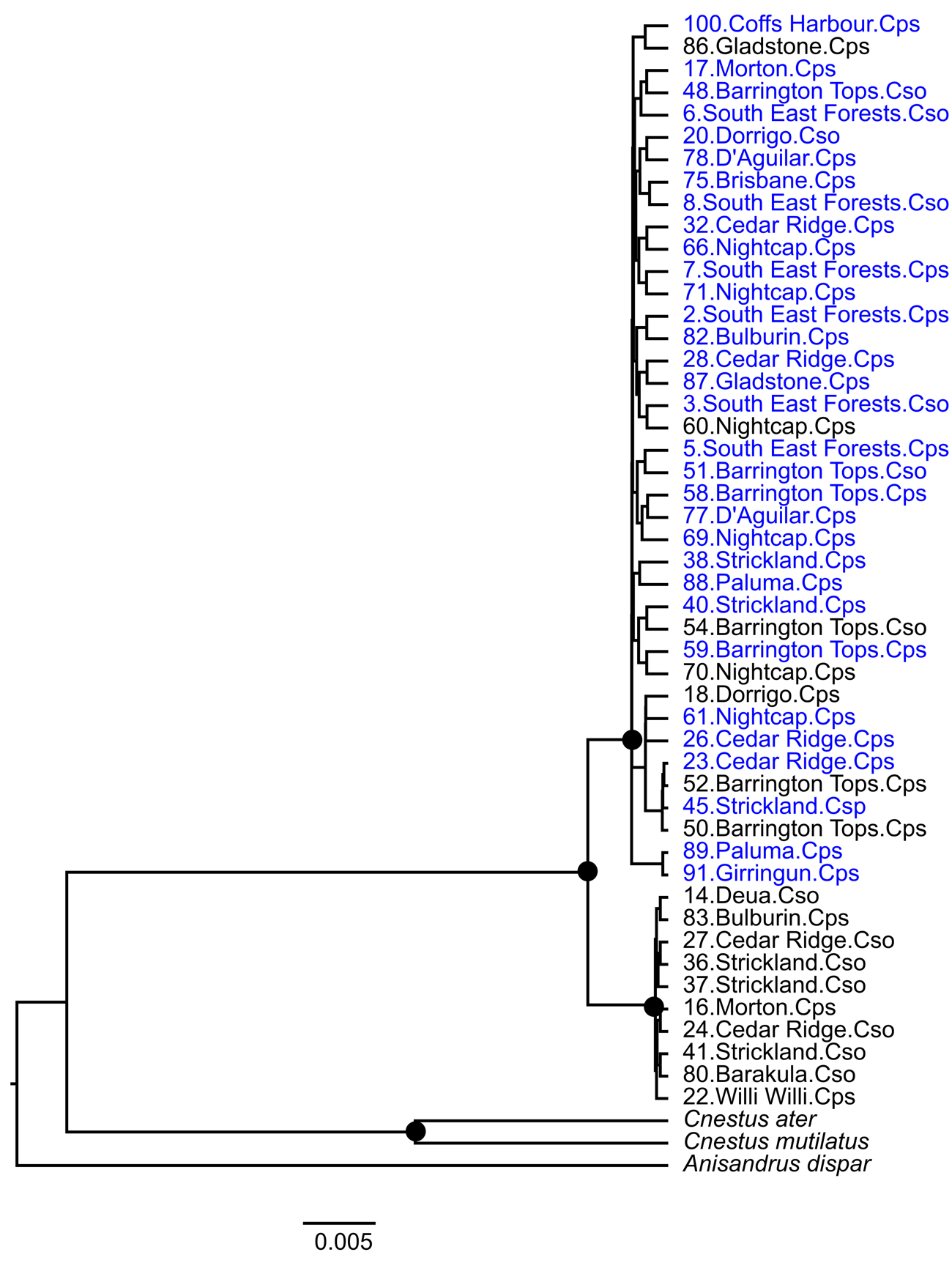
**Figure S2.** Phylogenetic tree of the partial *arginine kinase* gene (639 bp) of *Cnestus solidus* (Cso) and *C. pseudosolidus* (Cps) individuals collected throughout eastern Australia. *Wolbachia*-positive individuals are in blue. Support is provided at the nodes with black dots indicating >0.95 posterior probability and >95% bootstrap support. Scale bar indicates 0.005 substitutions per nucleotide position.


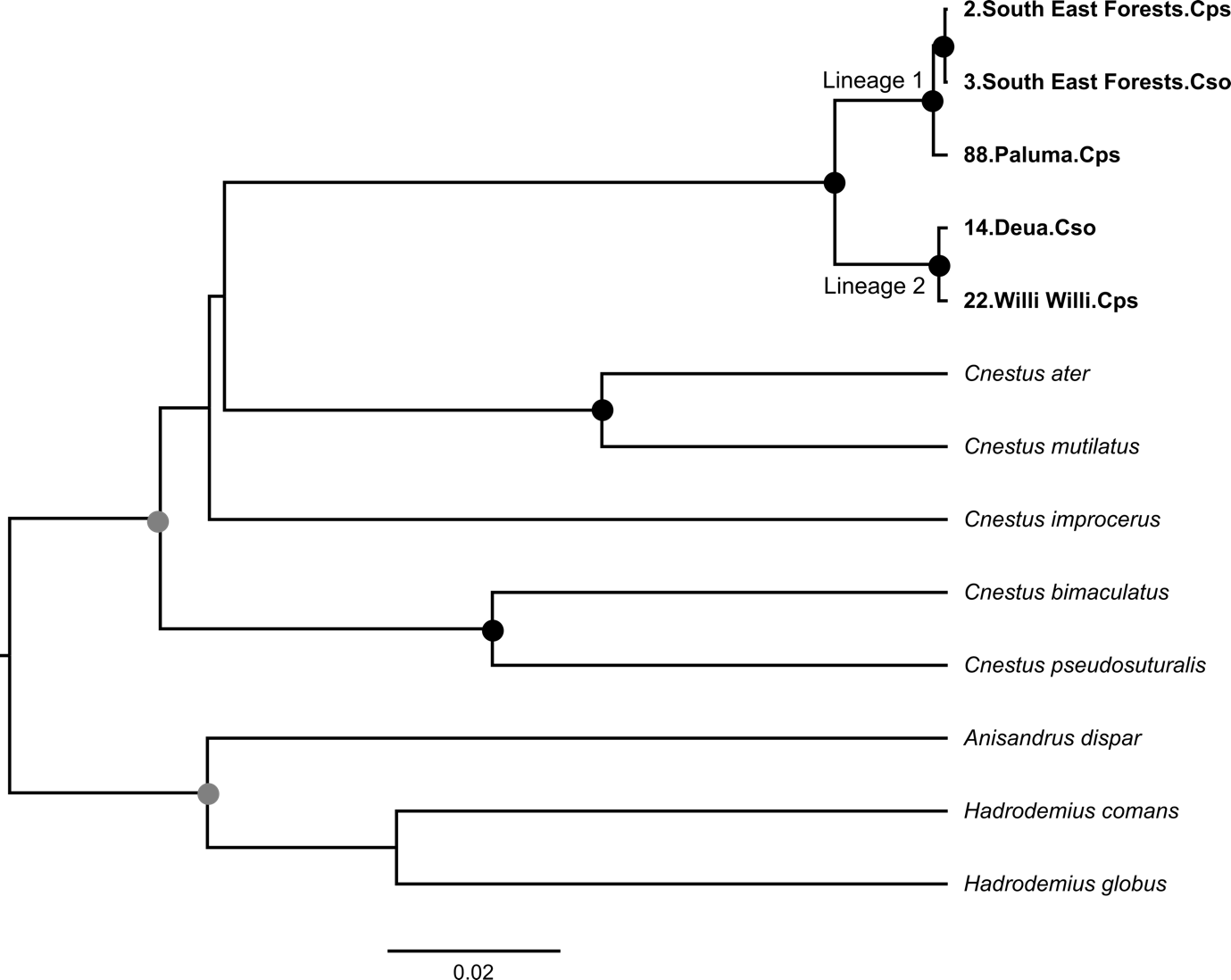
**Figure S3.** Phylogenetic tree of *Cnestus* using concatenated sequences (3069 bp) of the *cytochrome oxidase I*, *arginine kinase*, *carbamoyl-phosphate synthetase 2,* *elongation factor 1-alpha* and the *2D-3D segment of the nuclear 28S rRNA* genes. Individuals in bold are either *Cnestus solidus* lineages collected in this study and all other sequences were downloaded from GenBank (Table S6). Samples identified as *C. solidus* are labelled Cso, while those identified as *C. pseudosolidus* are labelled Cps. Support is given at the nodes with black dots indicating >0.95 posterior probability and >95% bootstrap support, while grey dots are >0.95 posterior probability alone. Scale bar indicates 0.02 substitutions per nucleotide position.

**
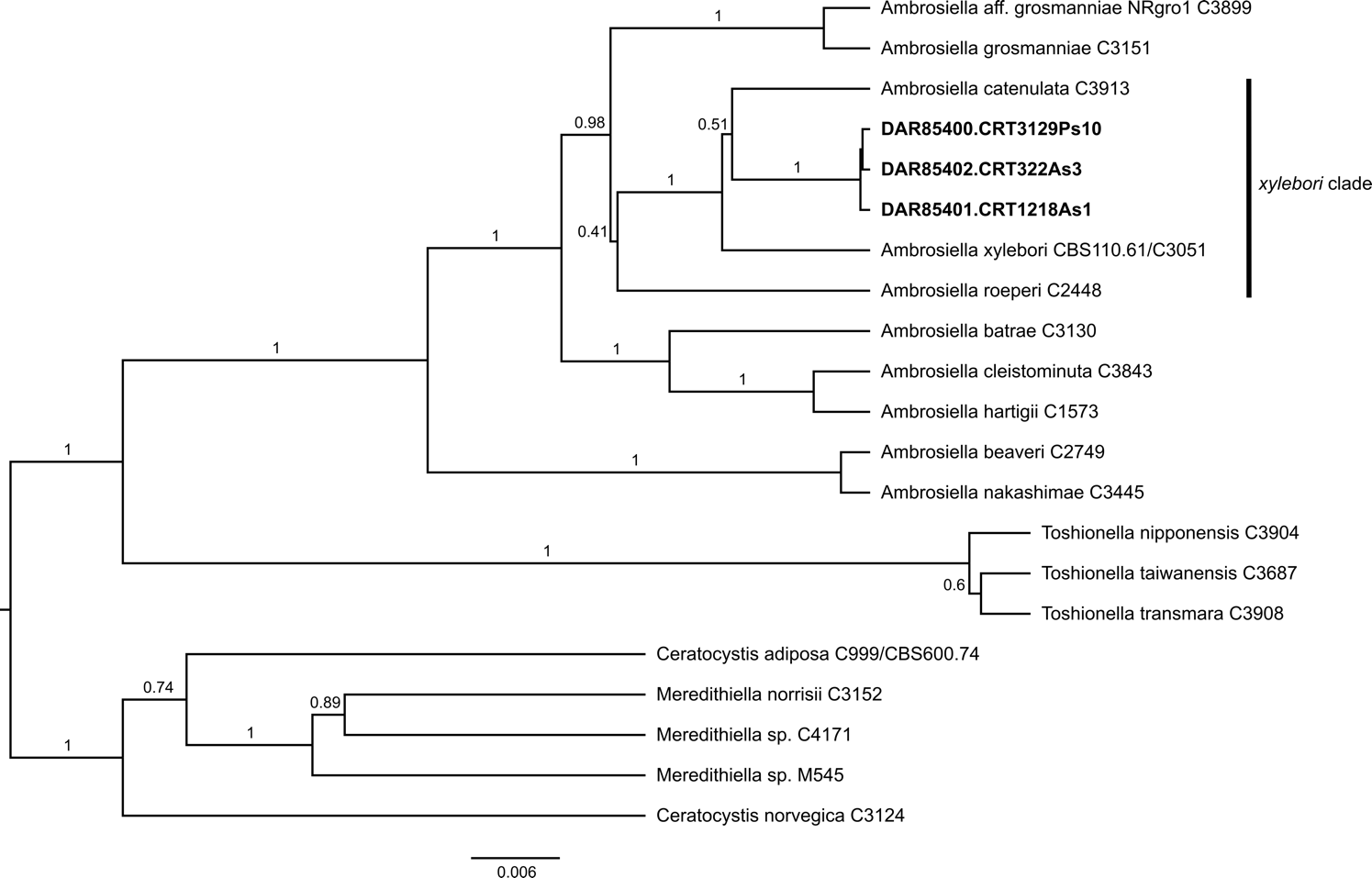
**

**Figure S4.** Bayesian phylogeny of *Ambrosiella*, *Toshionella* and outgroup taxa using concatenated sequences (3524 bp) of the 28S rRNA, 18S rRNA and *translation elongation factor 1-alpha* genes. Individuals in bold are *Ambrosiella* isolate 1 (Amb1) cultures obtained in this study, while other sequences were downloaded from GenBank (Table S5). Support is given at the nodes with black dots indicating >0.95 posterior probability. Scale bar indicates 0.006 substitutions per nucleotide position.
